# The association of sleep health with epigenetic aging in early adolescence: exploratory insights from the Health Outcomes and Measures of the Environment study

**DOI:** 10.1093/sleepadvances/zpag090

**Published:** 2026-08-13

**Authors:** Smriti Maskey, Jennifer Arzu, Aimin Chen, Karl T Kelsey, Scott M Langevin, Kim M Cecil, Kimberly Yolton, Joseph M Braun, Clara G Sears

**Affiliations:** Christina Lee Brown Envirome Institute, University of Louisville School of Medicine, Louisville, KY, United States; Department of Epidemiology and Population Health Sciences, University of Louisville School of Public Health and Information Sciences, Louisville, KY, United States; Department of Epidemiology, School of Public Health, Brown University, Providence, RI, United States; Department of Biostatistics, Epidemiology and Informatics, University of Pennsylvania Perelman School of Medicine, Philadelphia, PA, United States; Department of Epidemiology, School of Public Health, Brown University, Providence, RI, United States; Larner College of Medicine, University of Vermont, Burlington, VT, United States; Department of Medicine, University of Vermont Cancer Center, Burlington, VT, United States; Department of Radiology, University of Cincinnati College of Medicine, Cincinnati, OH, United States; Department of Environmental and Public Health Sciences, University of Cincinnati College of Medicine, Cincinnati, OH, United States; Department of Pediatrics, Cincinnati Children’s Hospital Medical Center, University of Cincinnati College of Medicine, Cincinnati, OH, United States; Department of Environmental and Public Health Sciences, University of Cincinnati College of Medicine, Cincinnati, OH, United States; Department of Pediatrics, Cincinnati Children’s Hospital Medical Center, University of Cincinnati College of Medicine, Cincinnati, OH, United States; Department of Epidemiology, School of Public Health, Brown University, Providence, RI, United States; Christina Lee Brown Envirome Institute, University of Louisville School of Medicine, Louisville, KY, United States

**Keywords:** epigenetic age, epigenetic age acceleration, biological aging, DNA methylation, sleep, actigraphy

## Abstract

**Study Objectives:**

Sleep during adolescence may influence biological aging, yet few studies have examined this association using objective sleep measures. We examined the associations between accelerometry-based sleep characteristics and epigenetic age (EA), epigenetic age acceleration (EAA), and the pace of biological aging.

**Study Methods:**

For this preliminary analysis, we used data from the Health Outcomes and Measures of the Environment Study (Cincinnati, OH). At age 12 years (2016–2019), we used accelerometers to assess total sleep time, sleep efficiency, the number of awakenings, and sleep fragmentation. Using DNA methylation data at the same age, we estimated EA and EAA using the skinbloodHorvath, Hannum, and Wu clocks, and the pace of biological aging using DunedinPACE. We used multivariable linear regression to estimate covariate-adjusted associations between sleep and epigenetic aging. We also evaluated sex modification by including product interaction terms and examined sex-stratified models.

**Results:**

Among 138 adolescents (59 per cent female; mean age 12.29 ± 0.64 years), general trends suggested that poorer sleep quality was associated with higher EA and EAA. However, all effect estimates were imprecise, and confidence intervals (CIs) included the null. Notably, we found that sex modified these relationships: higher sleep fragmentation was associated with elevated EAA among males (Hannum *β* = 0.885, 95% CI: 0.132, 1.638; interaction *p* = .005), but not females (*β* = –0.325, 95% CI: –0.866, 0.217). These associations were consistent across first-generation clocks.

**Conclusions:**

Overall, we did not find consistent evidence of associations between sleep characteristics and epigenetic aging in this preliminary analysis. However, sex-stratified analyses suggested that poorer sleep quality may be associated with greater EAA among males.

## Graphical Abstract

**Graphical Abstract:**
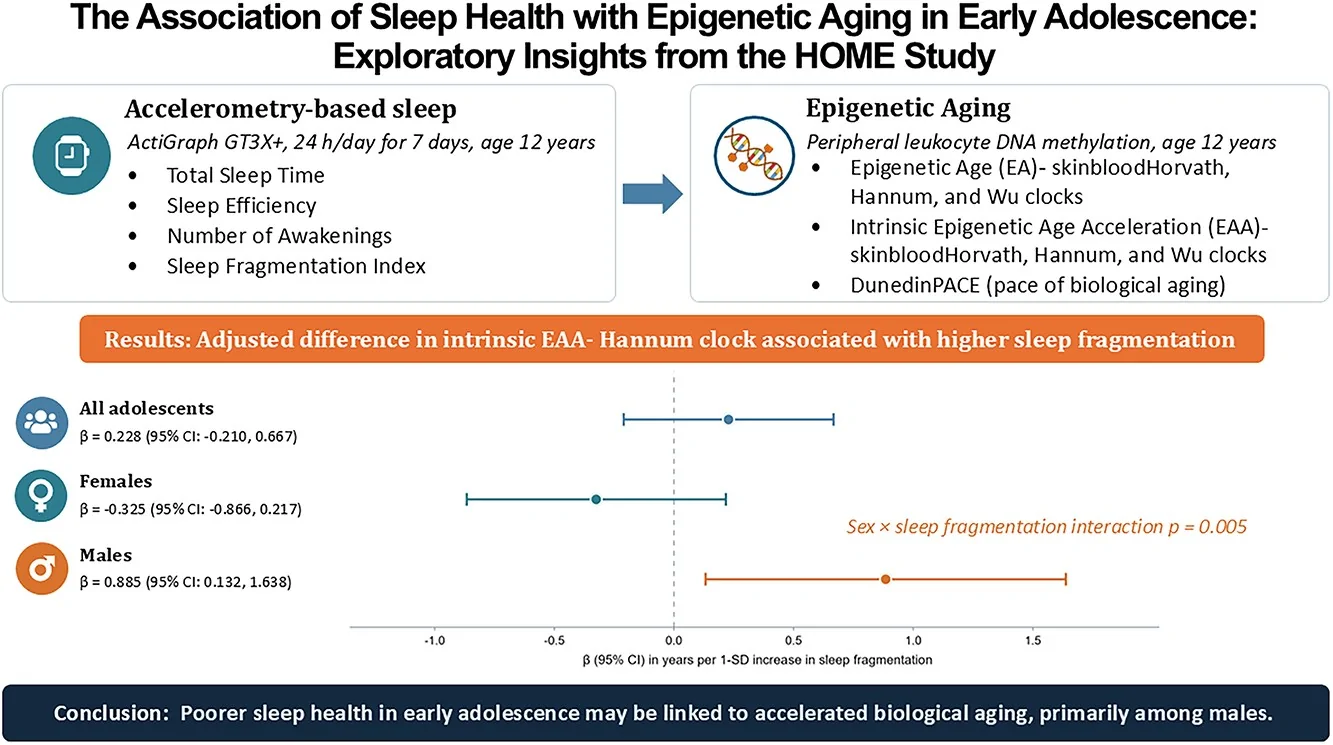

**Statement of Significance**

Sleep health during adolescence is increasingly recognized as critical for lifelong health, yet its link to biological aging measures remains poorly understood. This study examined the association between objective sleep measures and multiple novel biological aging metrics in early adolescence, a sensitive period of rapid development. We found some evidence suggesting that poorer sleep may be associated with accelerated biological aging, though the patterns were nuanced. Notably, these associations appeared primarily among males, suggesting a potential sex-specific vulnerability in how sleep becomes biologically embedded. Important knowledge gaps remain regarding how these early differences evolve over time and whether improving sleep during adolescence can alter long-term health trajectories, underscoring the need for longitudinal and interventional research.

## Introduction

Sleep quality and duration are crucial for optimal cognitive and physical development in childhood and early adolescence [1]. Inadequate sleep significantly disrupts the normal functioning of the hypothalamic–pituitary–adrenal (HPA) axis, which is crucial for regulating the body’s stress response and maintaining physiological balance [2]. This disruption can lead to prolonged activation of the HPA axis, resulting in irregular and elevated cortisol levels [3]. Chronic dysregulation of cortisol affects cellular processes, including metabolism and immune function, impairing the body’s ability to recover and regenerate during sleep [4, 5]. Over time, this disruption could contribute to biological aging by accelerating cellular damage and hindering restorative processes. Possible mechanisms include the impairment of DNA repair and increased oxidative stress, both of which are crucial for maintaining cellular integrity. Prolonged disruption of these processes may lead to epigenetic changes, such as altered DNA methylation (DNAm) patterns, which serve as biomarkers of biological aging. These changes are reflected in higher epigenetic age acceleration (EAA), a measure indicating that biological age surpasses chronological age [6]. Therefore, poor sleep quality and inadequate duration may accelerate biological aging by affecting key restorative processes, ultimately increasing the risk of age-related diseases such as cardiovascular disease, cognitive decline, and metabolic disorders [6–8].

Research has increasingly demonstrated a positive relationship between poor sleep health and accelerated epigenetic aging, although most evidence to date is derived from middle-aged and older adult populations [5, 9, 10]. Far fewer studies have evaluated these associations in younger cohorts. Carskadon et al. [11] conducted a prospective study of 12 first-year college women who completed daily online sleep diaries for 9 weeks and found no cross-sectional association between sleep duration or sleep regularity and EAA, as measured by the Horvath clock. However, shorter and less regular sleep was prospectively associated with accelerated EAA, whereas longer and more regular sleep was associated with decelerated EAA. In contrast, Balfour et al. [12] assessed parent-reported sleep trajectories from ages 5–17 and self-reported sleep problems at age 17 in 1192 adolescents and observed no association with EAA. However, the subjective reporting of the sleep measures could have influenced the results of this study, as individuals tend to systematically misestimate their sleep duration when compared with objective measures such as actigraphy [13].

Given these mixed findings and the limited availability of objective sleep assessments in prior research, including younger populations, the present study examined associations between actigraphy-measured sleep parameters and EAA in early adolescence. We focused on sleep efficiency, total sleep time, number of awakenings, and sleep fragmentation as key aspects of sleep continuity and duration that may contribute to variability in biological aging during this developmental period. This study aimed to evaluate whether these sleep characteristics are associated with accelerated biological aging or the pace of biological aging, thereby extending emerging work on sleep and epigenetic aging in earlier stages of the life course.

## Materials and methods

### Study design and participants

The data for this analysis came from the Health Outcomes and Measures of the Environment (HOME) Study, for which detailed descriptions have been published previously [14, 15]. Briefly, the HOME Study is a prospective pregnancy and birth cohort established to investigate how early-life environmental chemical exposures influence child health and development.

We recruited pregnant women living in the greater Cincinnati, Ohio, metropolitan area between March 2003 and January 2006 through nine local prenatal practices affiliated with three area hospitals. To be eligible, women had to be at least 18 years old, at 16 ± 3 weeks of gestation, living in a non-mobile, non-trailer home built before 1979, HIV-negative, not taking medications for seizures or thyroid disorders, and have no history of diabetes, bipolar disorder, schizophrenia, or cancer. We also required participants to plan to continue prenatal care and deliver at participating clinics and hospitals, and to remain in the study region for at least one year [14].

We enrolled a total of 468 pregnant women in the HOME Study. Of these, 67 dropped out during pregnancy, although 11 later re-enrolled at a follow-up visit. These participants gave birth to 420 children (398 singletons and 11 sets of twins) who completed at least one follow-up visit in childhood or adolescence. Between 2016 and 2019, at approximately 12 years of age (range 11–14 years), we conducted a comprehensive follow-up visit that included questionnaires, biospecimen collection, neuroimaging, dual-energy X-ray absorptiometry, and other assessments to examine the long-term impacts of early environmental exposures on adolescent health [15].

For this analysis, we included only those adolescent participants who completed the 12-year follow-up visit (*n* = 256). We further restricted the sample to singleton children (*n* = 242). We excluded participants who were missing (1) preprocessed DNA methylation data needed to estimate epigenetic age (EA) at age 12 years (*n* = 54), (2) valid ActiGraph data or who did not have sufficient wear time (*n* = 45), and (3) covariate data required for the analysis (*n* = 5). After these exclusions, the final sample for our complete case analysis included 138 participants (Figure S1). Among these participants, no caregivers reported any physician-diagnosed sleep disorders.

### Ethical considerations of the HOME study

The HOME Study protocol, including all recruitment materials, consent forms, and data collection instruments, was reviewed and approved by the Institutional Review Boards (IRBs) of Cincinnati’s Children’s Hospital Medical Center (CCHMC) and each participating delivery hospital. The U.S. Centers for Disease Control and Prevention (CDC) relied on the CCHMC IRB [15]. During the initial prenatal visit, trained research assistants met individually with each participant to explain the study objectives, procedures, potential risks and benefits, and the measures taken to ensure confidentiality. We obtained written informed consent from all mothers for both their own participation and that of their children. At the 12-year follow-up visit, we obtained written informed assent from the adolescents after thoroughly explaining the study procedures and answering any questions [15]. The secondary analysis of HOME Study data was approved by the IRB at the University of Louisville.

### Sleep assessment (exposure)

During 12-year study visits at the research site, we provided the participants with an ActiGraph GT3X+ accelerometer (Actigraph, LLC, Pensacola, FL) and directed them to wear it on their left wrist 24 hours a day for seven days [15]. Upon receiving the devices back through the mail, we utilized ActiLife software (version 6.13.4) to extract the data. Initially, we conducted a visual inspection of each participant's data to identify periods of non-wear [16]. We employed the Sadeh et al. [17] algorithm to categorize 1-minute epochs as either sleep or wake, which was specifically designed for wrist-worn devices. We established sleep onset as the beginning of five consecutive epochs classified as sleep and defined the sleep period's end as the start of 15 consecutive minutes of wakefulness following sleep onset. During our visual assessment of each sleep period, we merged sleep periods with end/start times within 60 minutes. We determined sleep duration by calculating the total sleep time between sleep onset and offset. The primary nocturnal sleep periods were required to begin between 19:00 and 05:59 and last for at least 160 minutes [18]. Our analysis included participants who had a minimum of four valid nocturnal sleep periods that did not coincide with non-wear time, comprising at least three valid weeknights and one weekend night [18, 19]. We calculated the average number of awakenings per sleep period and defined sleep efficiency as the average percentage of sleep time (calculated by dividing the number of minutes spent sleeping by the total duration between sleep onset and offset) [20]. Furthermore, we used the average Total Sleep Fragmentation Index score, which quantifies restlessness as a percentage, with higher scores indicating increased movement and sleep disruption [21]. This index combines the movement index (the percentage of scored epochs with more than one activity count) and the sleep fragmentation index (the percentage of 1-minute sleep epochs relative to all sleep periods between onset and offset). These objectively derived dimensions (duration, continuity, and efficiency) capture core aspects of sleep health relevant to adolescent development and have been linked to epigenetic aging outcomes in prior studies [22].

### Epigenetic age (outcome)

We used preprocessed DNAm data to estimate EA at 12 years using multiple epigenetic clocks: Horvath skin and blood (skinbloodHorvath) [23], Hannum [24], Wu [25], and the more recently developed DunedinPACE [26], which assesses the pace of biological aging. We selected these clocks based on their widespread use in the literature, allowing for comparison with other studies, and because they were trained on tissue types consistent with our cohort’s biospecimens [23–26]. The characteristics of these clocks have been described in previous reports [23–26]. Briefly, the first-generation clocks (skinbloodHorvath, Hannum, and Wu) estimate age by applying elastic net regression models to DNAm data to predict chronological age based on CpG site patterns [23–25]. In contrast, DunedinPACE, a third-generation clock, estimates the pace of biological aging based on longitudinal changes in 19 biomarkers measured from early to mid-adulthood [26]. The different epigenetic clocks exhibited considerable variation in their design and the demographic populations used for data training, with minimal overlap in the chosen CpGs across the clocks [27]. By leveraging multiple epigenetic clocks, we aimed to assess the robustness of associations between sleep health and biological aging across diverse algorithmic approaches and training populations in early adolescence.

We used the *methylclock* [28] R package (v1.18.0) to compute EA and corresponding EAA metrics. EAA was calculated as the residual from regressing EA on chronological age, where positive values indicate accelerated biological aging [27]. Recognizing the influence of cell type composition on EA, we also derived intrinsic EAA measures by adjusting for estimated cell-type proportions. We estimated these proportions using reference-based methods developed by Salas et al. [29] for adult peripheral blood implemented in methylclock [28].

To estimate the pace of biological aging, defined as the average rate of biological aging per chronological year, we applied the DunedinPACE algorithm. We computed an intrinsic pace measure using a linear regression model adjusted for estimated cell-type proportions, excluding neutrophils due to concerns about multicollinearity [27]. For the first-generation clocks, we addressed missing CpG probes using *k*-nearest neighbors imputation (*k* = 10) within methylclock, consistent with the methods used for the Wu and Hannum clocks. For DunedinPACE, we applied mean imputation using the PACEProjector function from the DunedinPACE [26] R package (v0.99.0) [27].

### Covariates

Trained research staff conducted standardized interviews with caregivers during the 12-year study visit to collect information on maternal education and the adolescent’s racial background. Most participants reported their child’s race as white or black/African American. Participants identifying as Asian/Pacific Islander or American Indian were grouped into a single “Other/Multiracial” category due to limited sample size (*n* = 12). We abstracted infant sex from hospital medical charts at birth.

To assess diet quality, trained staff administered three 24-hour dietary recalls during and after the 12-year visit. To capture the day-to-day variability, recalls were strictly scheduled to include two weekdays and one weekend. We used the Nutrition Data System for Research software and food database (University of Minnesota) to calculate Healthy Eating Index (HEI) scores at the 12-year visit, which measure alignment with federal dietary guidelines, with higher scores indicating better diet quality [30, 31].

Adolescents self-reported their pubertal development at the 12-year visit by selecting images that best represented their stage of pubic hair growth, as defined by Tanner staging [32]. Stage 1 reflects a prepubertal state with no pubic hair; stage 2 marks the onset of puberty with sparse, lightly pigmented hair; stages 3 and 4 show progressively darker and coarser hair; and stage 5 indicates full adult pubic hair development.

We estimated adolescent serum cotinine at the 12-year visit to assess concurrent secondhand smoke exposure, given established evidence linking objective measures of recent exposure to significant sleep disruption in youth [33]. We categorized the serum cotinine variable in tertiles for the descriptive summarization of the sample population (Table 1), but used the continuous values in the regression models. We assessed moderate-to-vigorous physical activity (MVPA) using data from a wrist-worn ActiGraph GT3X+ device, applying Chandler et al.’s [34] youth-specific cut-points. The average daily duration of MVPA (in minutes) was calculated across seven valid days, defined as having ≥10 hours of wear time per day [34, 35].

**Table 1:** Participant characteristics and mean ± SD values of covariates by sleep outcomes in complete case analysis (*n* = 138, the HOME study, cincinnati, OH, 2003–2019)

Category	Total [n (%)]	Sleep efficiency (%)	Total sleep time (min)	Number of awakenings (n)	Sleep fragmentation index (%)
**Overall**	**138 (100)**	**88 ± 4**	**425 ± 55**	**20 ± 6**	**21 ± 6**
**Adolescent sex**					
Female	81 (59)	89 ± 4	434 ± 56	19 ± 6	21 ± 6
Male	57 (41)	87 ± 5	414 ± 52	21 ± 6	22 ± 6
**Adolescent race**					
White	80 (58)	88 ± 4	440 ± 50	22 ± 6	21 ± 5
Black or African American	46 (33)	90 ± 4	409 ± 56	17 ± 5	20 ± 6
Other/Multiracial^*^	12 (9)	86 ± 4	394 ± 58	21 ± 5	24 ± 5
**Age (Years, in tertiles)**					
11.05 to 12.03	50 (36)	89 ± 4	428 ± 49	19 ± 6	20 ± 5
12.03 to 12.65	50 (36)	87 ± 5	423 ± 59	21 ± 6	22 ± 6
12.65 to 14.09	38 (28)	88 ± 4	425 ± 59	20 ± 5	21 ± 5
**Mother’s education**					
College graduate or above	73 (53)	88 ± 4	441 ± 50	21 ± 5	22 ± 6
High school and some college	57 (41)	89 ± 5	413 ± 52	18 ± 6	20 ± 6
Less than high school	8 (6)	87 ± 5	366 ± 65	18 ± 3	23 ± 5
**Asthma diagnosis**					
No	107 (78)	88 ± 4	421 ± 56	20 ± 6	21 ± 6
Yes	31 (22)	89 ± 4	440 ± 52	19 ± 6	21 ± 5
**ADHD diagnosis**					
No	113 (82)	88 ± 4	429 ± 56	20 ± 6	21 ± 5
Yes	25 (18)	89 ± 5	408 ± 47	17 ± 6	20 ± 6
**Adolescent serum cotinine concentration (ng/mL)**					
< 0.04	46 (33)	88 ± 4	430 ± 51	21 ± 5	22 ± 6
0.04 to 0.08	49 (36)	88 ± 4	436 ± 59	22 ± 6	21 ± 5
> 0.08	43 (31)	90 ± 4	409 ± 53	17 ± 6	20 ± 6
**BMI** ^†^					
Underweight	3 (2)	84 ± 6	344 ± 76	20 ± 3	23 ± 3
Normal weight	83 (60)	89 ± 4	434 ± 54	20 ± 6	21 ± 5
Overweight	43 (31)	88 ± 5	416 ± 51	20 ± 7	22 ± 6
Obesity	9 (7)	90 ± 3	420 ± 55	16 ± 4	17 ± 6
**Anxiety** ^**‡**^					
Normal (<60)	117 (85)	88 ± 4	428 ± 50	20 ± 6	21 ± 6
Elevated (60 and above)	21 (15)	89 ± 6	410 ± 76	18 ± 7	21 ± 6
**Physical activity** **^§^**					
Inactive	17 (12)	88 ± 3	429 ± 65	20 ± 5	20 ± 5
Meets minimum Recommendation	51 (37)	88 ± 4	418 ± 60	19 ± 6	22 ± 5
Above recommendation	44 (32)	89 ± 4	438 ± 47	20 ± 6	21 ± 5
Highly active	26 (19)	88 ± 4	417 ± 50	21 ± 6	22 ± 7
**HEI** ^**||**^					
< 39.84	45 (33)	88 ± 4	434 ± 50	20 ± 6	22 ± 5
39.84 to 48.88	44 (32)	89 ± 5	421 ± 66	19 ± 7	21 ± 7
> 48.88	49 (36)	88 ± 3	422 ± 49	20 ± 5	21 ± 5

Abbreviations: HOME, Health Outcomes and Measures of the Environment; SD, standard deviation.

^*^Other/Multiracial includes participants identifying as Asian/Pacific Islander or American Indian

^†^BMI was measured as a *z*-score. Categories are defined as: Underweight (*z* < –2), Normal weight (-2 ≤ *z* < 1), Overweight (1 ≤ *z* < 2), and Obesity (*z* ≥ 2).

^‡^Anxiety level was assessed using Total Scores from the Spence Children’s Anxiety Scale (range: 1–100). Elevated anxiety was defined as a score ≥ 60; normal anxiety was defined as a score < 60.

^§^Physical activity represents the average daily minutes of moderate-to-vigorous physical activity at age 12, measured by wrist-worn accelerometers. Categories are defined as: inactive (< 60 min/day), meets minimum recommendation (60–90 min/day), above recommendation (90–120 min/day), and highly active (≥ 120 min/day).

^||^HEI scores were based on the HEI-2010 (total possible score: 100), derived from food frequency questionnaire data. Boldface does not signify statistical significance, effect size, or any other attribute of the underlying data.

We calculated adolescent body mass index (BMI) *z*-scores based on measured height and weight, using the CDC growth charts for standardization [15]. Physician-diagnosed asthma and attention-deficit/hyperactivity disorder (ADHD) were reported by their caregivers at 12-year visit and coded as binary variables [15]. Adolescent anxiety symptoms were measured continuously using the 44-item self-reported Spence Children’s Anxiety Scale, which assesses six domains of anxiety in youth [36]. For regression models, we used the Total Anxiety Score to capture overall symptom burden; for the sample description (Table 1), scores were dichotomized as elevated (≥ 60) versus normal (< 60) anxiety.

To identify covariates and potential confounding variables relevant to sleep and EA at 12 years, we relied on prior literature and a directed acyclic graph (DAG) (Figure S2). Covariates included sociodemographic factors such as adolescent sex (male or female), age at sleep assessment (years), race/ethnicity (white, black/African American, Other/Multiracial), and maternal education (less than high school, high school, some college, college graduate or above). Additional covariates included adolescent serum cotinine concentrations (ng/mL), HEI score (dimensionless), ADHD and asthma diagnoses (yes/no), adolescent BMI *z*-score (dimensionless), and adolescent anxiety level (elevated/normal). Together, these covariates represent a range of sociodemographic, behavioral, and clinical factors that may influence both sleep patterns and epigenetic aging in early adolescence.

### Statistical analysis

**Figure 1:**
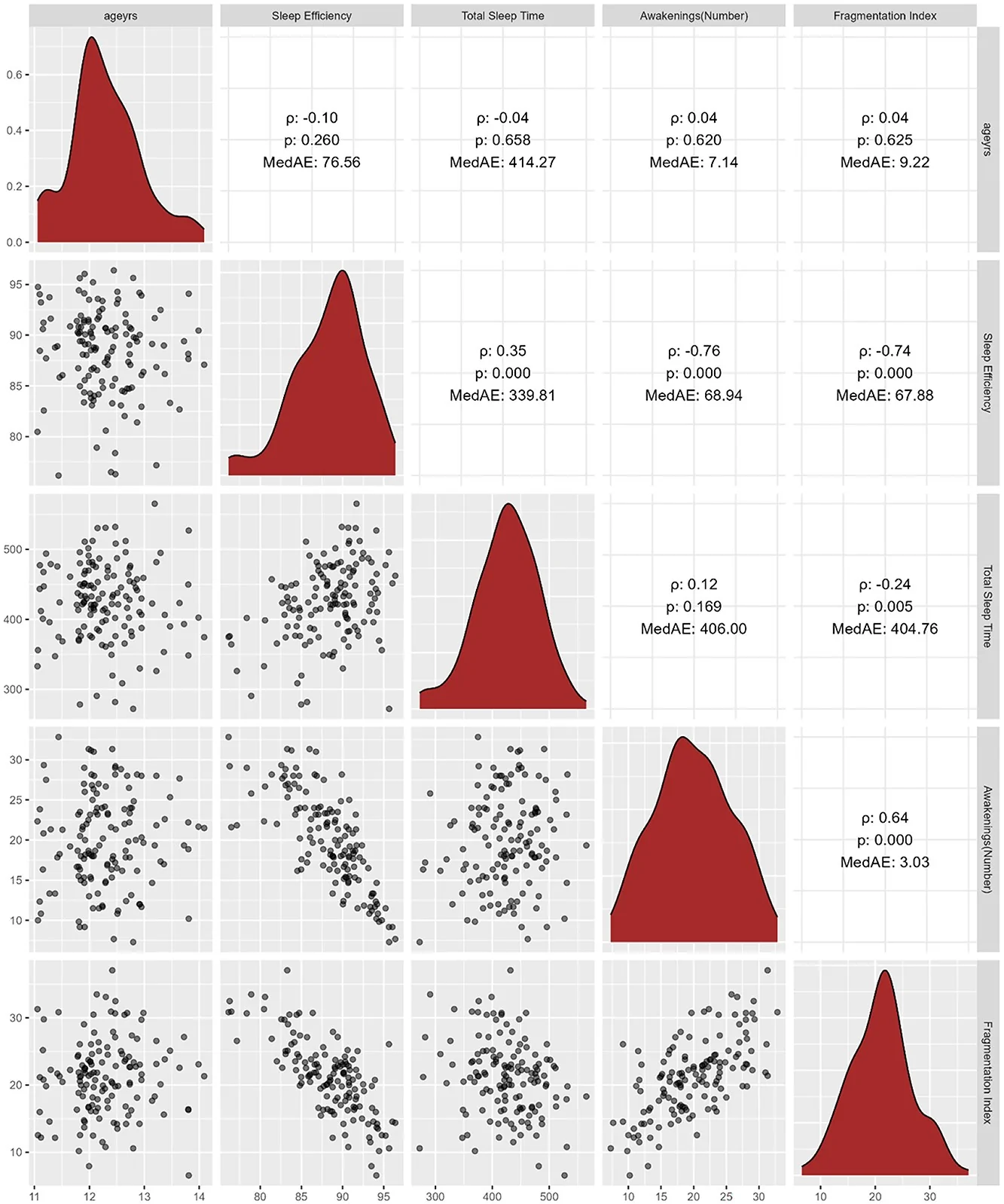
Pearson correlation of chronological age and sleep outcomes among singleton adolescents from the HOME Study (*n* = 138).

We summarized the demographic, clinical, and behavioral characteristics (mean and standard deviation) of mothers and adolescents in the study sample stratified by sleep outcomes (Table 1). We assessed differences in characteristics between included and excluded participants using Pearson’s chi-squared test, Fisher’s exact test for categorical variables, and the Wilcoxon rank-sum test for continuous variables (Table S2). We used Pearson correlation coefficients to examine the relationship between chronological age and sleep measures (Figure 1).

To estimate the association between sleep measures and EA, EAA, and the pace of biological aging at 12 years, we fit separate multivariable linear regression models with each sleep measure as the predictor and EA, EAA, and the pace of biological aging as outcomes. We applied partial post-hoc standardization by rescaling the sleep exposure coefficients using their standard deviations, allowing comparability across metrics while retaining the original scale of the outcomes.

All models were adjusted for adolescent serum cotinine (continuous, ng/mL), child sex (male/female), adolescent race/ethnicity (white, black or African American, Other/Multiracial), maternal education (less than high school, high school or some college, college graduate or above), asthma diagnosis (yes/no), ADHD diagnosis (yes/no), adolescent age at sleep assessment (continuous, years), adolescent BMI *z*-score (continuous), moderate-to-vigorous physical activity (continuous, minutes per day), HEI score (continuous), and Spence Children’s Anxiety Scale score (continuous). We excluded pubertal development in primary models because the Tanner stage may lie on the causal pathway between sleep and epigenetic aging, and adjusting for it could result in overadjustment bias (Figure S2) [37].

Intrinsic EA acceleration measures based on first-generation clocks (Wu, Hannum, and skinbloodHorvath) and the pace of biological aging (DunedinPACE) were previously adjusted for estimated blood cell-type proportions [27, 29, 38]. Therefore, we did not include cell-type proportions as covariates in our regression models. Because prior literature has reported nonlinear associations between total sleep time and health outcomes, we additionally conducted exploratory spline analyses for total sleep time only; full methods and results are presented in the Supplement (Figure S3 and Table S1).

In secondary analyses, we examined whether child sex modified the association between adolescent sleep and EA, EAA, and the pace of biological aging at 12 years. We prioritized sex-stratified analyses to account for established biological and behavioral dimorphism. Biologically, hormonal changes during pubertal development may modulate both sleep architecture and DNA methylation pathways, potentially driving sex-specific aging trajectories [39–42]. Furthermore, distinct behavioral sleep patterns and environmental sensitivities during adolescence may differentially influence the biological embedding of sleep in males and females [37, 43]. For these interaction models, we considered a *p*-value < .05 as suggestive of effect measure modification. We also conducted sex-stratified analyses using the same covariate-adjusted models described above, barring sex.

### Sensitivity analysis

We conducted several sensitivity analyses to assess the robustness of our findings to residual confounding and missing data. First, we repeated our main analyses after removing statistical outliers identified using Cook’s distance, a standard diagnostic for detecting influential observations in linear regression [44]. The number of outliers varied across models, ranging from 5 to 13 (3.62 per cent to 9.42 per cent of the analytic sample), depending on the distribution of the exposure-outcome combination. Second, we re-estimated our models after excluding BMI *z*-scores. While BMI was included in our primary analyses as a potential confounder, it may also lie on the causal pathway between sleep and epigenetic aging through mechanisms such as metabolic dysregulation and inflammation. Thus, excluding BMI allowed us to evaluate the potential for overadjustment bias [45, 46]. Finally, we adjusted for pubertal stage in sex-stratified analyses, as pubertal stage could be related to sleep characteristics and EA.

## Results

### Participant characteristics

The complete case analytic sample comprised of 138 adolescents, with a mean age of 12.29 ± 0.64 years. Among the study participants, 59 per cent were female, 58 per cent were white, 33 per cent self-identified as black or African American, and 9 per cent as Other or Multiracial. Overall, the mean sleep efficiency was 88 per cent ± 4 per cent, the mean total sleep time was 425 ± 55 minutes, the mean number of awakenings was 20 ± 6, and the mean sleep fragmentation index was 21 per cent ± 5 per cent (Table 1).

Across sociodemographic subgroups, mean total sleep time was 434 ± 56 minutes among females and 414 ± 52 minutes among males. The mean total sleep time was 440 ± 50 minutes among white adolescents, 409 ± 56 minutes among black or African American adolescents, and 394 ± 58 minutes among those identifying as Other or Multiracial. Across maternal education categories, mean total sleep time ranged from 366 ± 65 minutes to 441 ± 50 minutes, with sleep efficiency averaging between 88 per cent and 89 per cent. The mean total sleep time across cotinine exposure and physical activity categories ranged from 409 ± 53 minutes to 438 ± 47 minutes, and sleep efficiency remained consistent across both, averaging between 88 per cent and 90 per cent. Across BMI categories, mean total sleep time ranged from 344 ± 76 minutes to 434 ± 54 minutes, though the underweight subgroup comprised only three participants and should be interpreted with caution.

The mean total sleep time was 440 ± 52 minutes among adolescents with an asthma diagnosis and 421 ± 56 minutes among those without. Among adolescents with an ADHD diagnosis, the mean total sleep time was 408 ± 47 minutes, and 410 ± 76 minutes among those with elevated anxiety scores. Across HEI tertiles, the mean total sleep time ranged from 421 ± 66 minutes to 434 ± 50 minutes. Sleep efficiency remained largely consistent across all subgroups, averaging between 86 per cent and 90 per cent, and detailed distributions of all sleep metrics are presented in Table 1.

Included and excluded participants showed similar characteristics at the 12-year visit, though the included group had a slightly higher proportion of overweight adolescents (Table S2). Compared with white adolescents, black or African American participants had lower mean EAA across the Wu (−0.37 ± 0.82 vs. 0.11 ± 0.89), skinbloodHorvath (−0.54 ± 2.76 vs. 0.12 ± 2.81), and Hannum clocks (−0.83 ± 2.72 vs. 0.08 ± 2.64). In contrast, their mean DunedinPACE values were higher (0.41 ± 1.22 vs. –0.26 ± 0.76). Adolescents whose mothers had less than a high school education exhibited higher average EAA on the skinbloodHorvath (2.54 ± 4.10) and Hannum clocks (1.83 ± 2.94) relative to those whose mothers completed college (0.07 ± 2.60 and –0.05 ± 2.69, respectively). ADHD status also appeared to align with differences in epigenetic aging, with lower mean EAA among adolescents without ADHD on the Wu (0.01 ± 0.88 vs. –0.40 ± 0.76) and skinbloodHorvath clocks (0.15 ± 2.81 vs. −1.32 ± 1.89). Higher serum cotinine concentrations corresponded to higher mean DunedinPACE values (>0.08 ng/mL: 0.21 ± 1.20) compared with the lowest exposure group (<0.04 ng/mL: –0.17 ± 0.94) (Table S3).

### Correlation between chronological age and sleep measures

Chronological age was not significantly correlated with any sleep metric (all *p* > .25; Figure 1). As expected, strong inverse correlations were observed between sleep efficiency and both the number of awakenings (*r* = −0.76, *p* < .001) and the fragmentation index (*r* = −0.74, *p* < .001), indicating that lower sleep efficiency is associated with more disrupted sleep. Total sleep time was positively correlated with sleep efficiency (*r* = 0.35, *p* < .001) and modestly inversely associated with fragmentation (*r* = −0.24, *p* = .005). These patterns support the internal validity and coherence of the objective sleep measures used in this study.

### Adjusted associations of sleep measures with epigenetic age at 12 years of age

**Figure 2:**
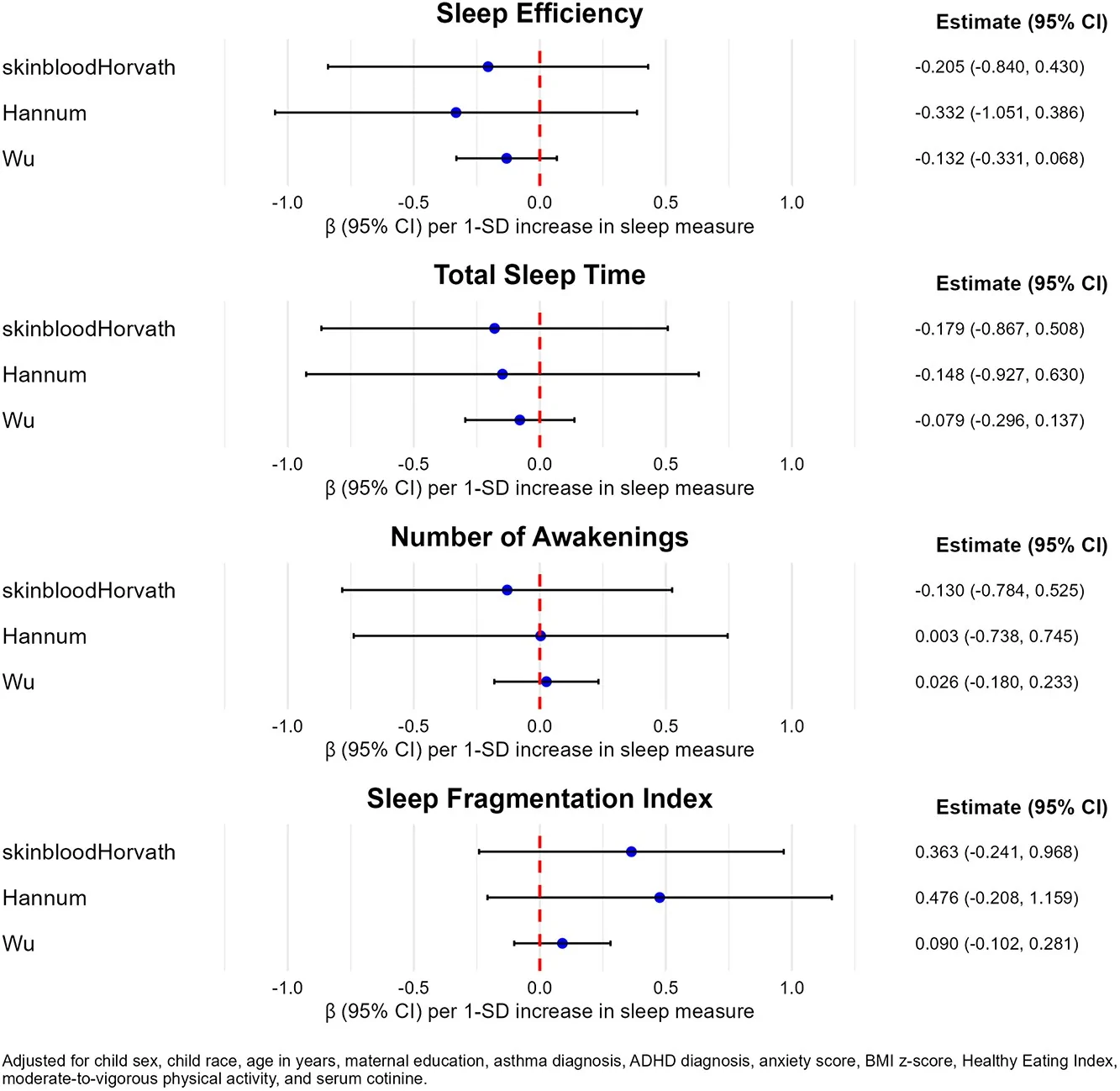
Adjusted difference (95% CI) in epigenetic clocks per standard deviation increase in sleep measures among adolescents (the HOME Study, Cincinnati, OH, 2003–2019, *n* = 138). Models were adjusted for adolescent age, sex, race, maternal education, asthma diagnosis, ADHD diagnosis, anxiety symptoms, serum cotinine concentrations, physical activity, BMI, and HEI-2010 scores.

In covariate-adjusted models, we observed largely null associations between actigraphy-derived sleep measures and EA estimated from first-generation clocks (skinbloodHorvath, Hannum, and Wu; Figure 2). General trends suggested that sleep efficiency and total sleep time were negatively associated with EA across clocks, while the sleep fragmentation index was positively associated with these outcomes. However, effect sizes were small, and confidence intervals (CIs) were wide and included the null for all analyses.

### Adjusted associations of sleep measures with epigenetic age acceleration and pace of biological aging at 12 years of age

**Figure 3:**
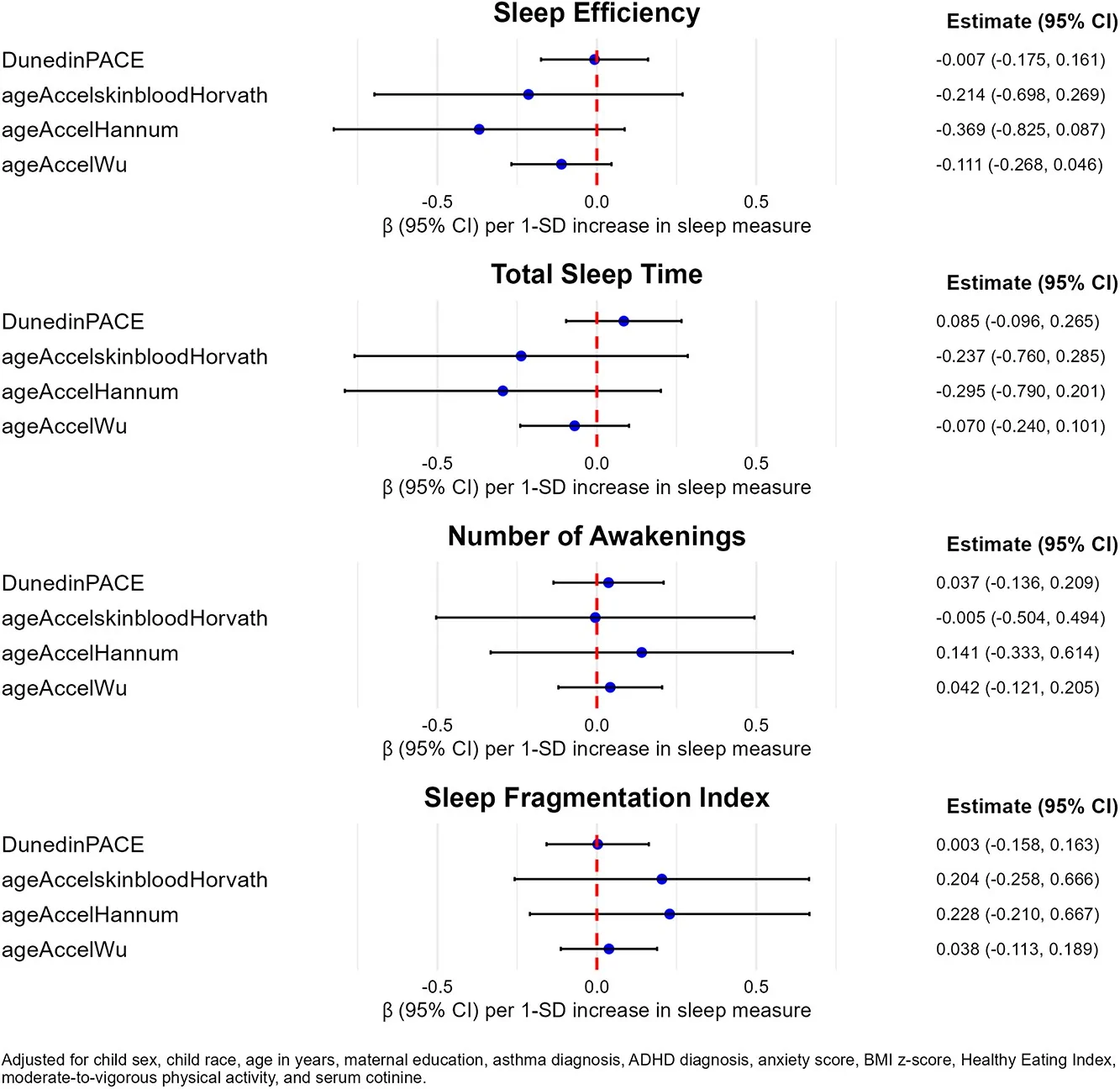
Adjusted difference (95% CI) in EAA and pace of biological aging per standard deviation increase in sleep measures among adolescents (the HOME study, Cincinnati, OH, 2003–2019, *n* = 138). DunedinPACE: Intrinsic pace of biological aging. ageAccelskinbloodHorvath: Intrinsic EAA, calculated for the skinbloodHorvath clock. ageAccelHannum: Intrinsic EAA, calculated for the Hannum clock. ageAccelWu: Intrinsic EAA, calculated for the Wu clock. Models were adjusted for adolescent age, sex, race, maternal education, asthma. diagnosis, ADHD diagnosis, anxiety symptoms, serum cotinine concentrations, physical activity, BMI *z*-score, and HEI-2010 scores.

In covariate-adjusted models, we observed largely null associations between actigraphy-derived sleep measures and EA acceleration estimated from the first-generation clocks (skinbloodHorvath, Hannum, and Wu) and the pace of biological aging (DunedinPACE) (Figure 3). We found general trends suggesting that higher sleep efficiency and longer total sleep time were inversely associated with EAA measures. However, effect sizes were small, and CIs included the null in all analyses.

### Sex differences in the association of sleep measures with EA, EAA, and pace of biological aging at 12 years of age

**Figure 4:**
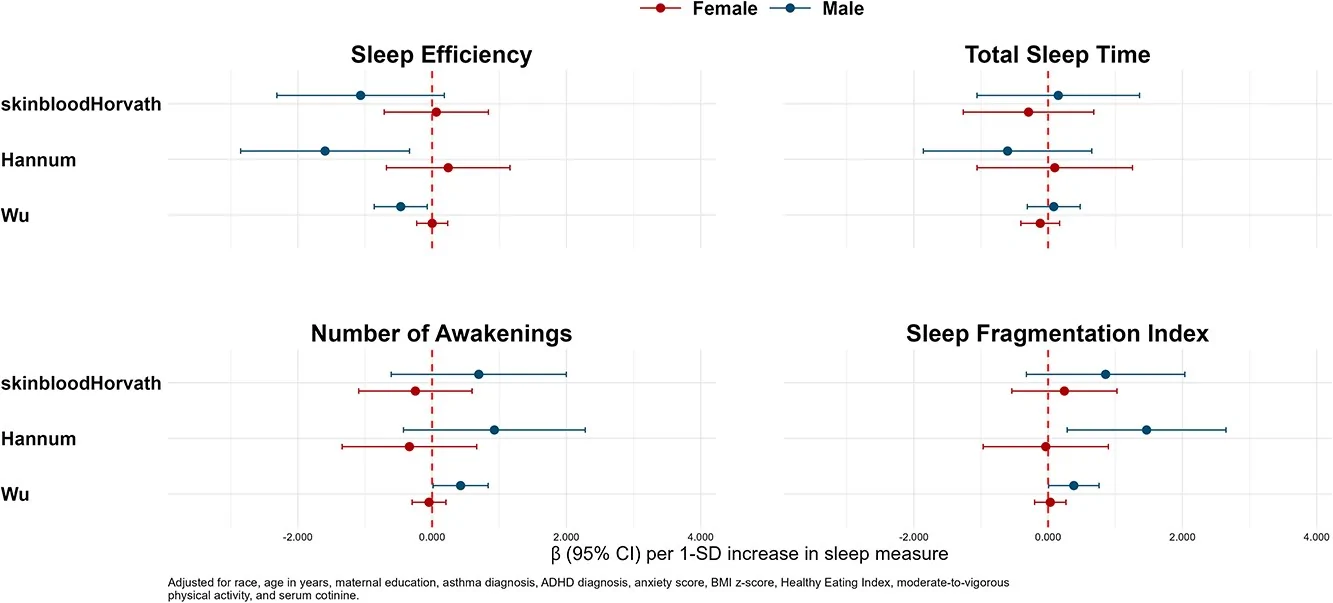
Adjusted difference in EA clocks per standard deviation increase in sleep measures when stratifying by sex (the HOME Study, Cincinnati, OH, 2003–2019, *n* = 138). Models were adjusted for adolescent age, race, maternal education, asthma diagnosis, ADHD diagnosis, anxiety symptoms, serum cotinine concentrations, physical activity, BMI *z*-score, and HEI-2010 scores.

**Figure 5:**
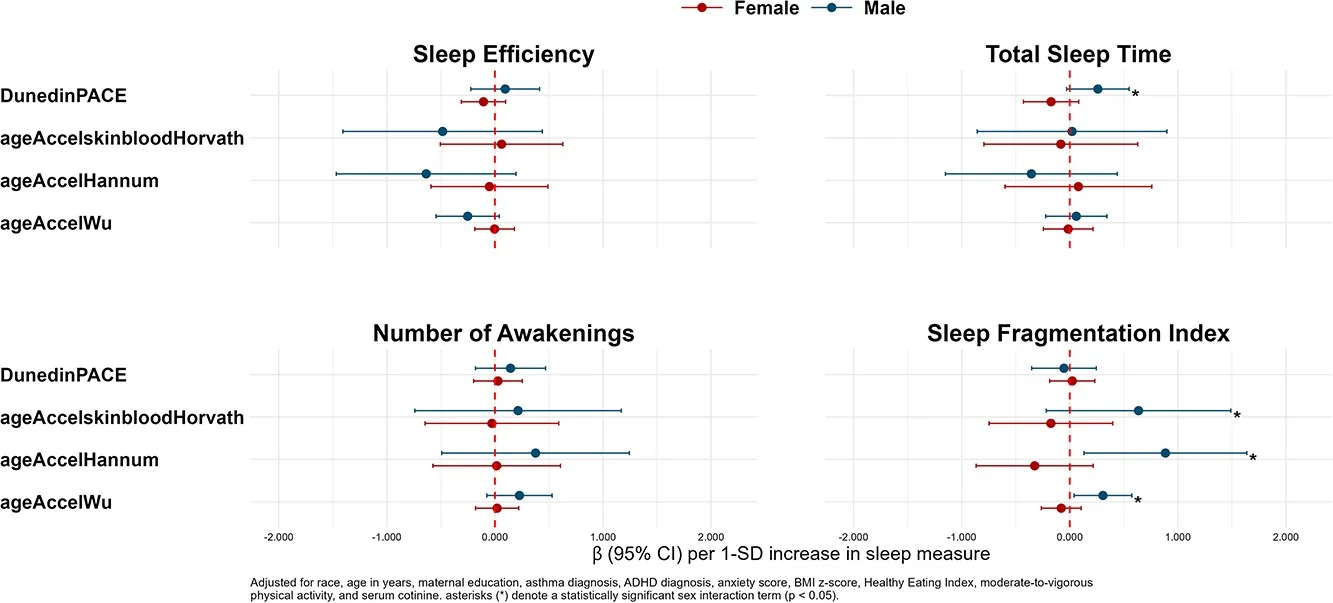
Adjusted difference in EAAs and pace of biological aging per standard deviation increase in sleep measures when stratifying by sex (the HOME study, Cincinnati, OH, 2003–2019, *n* = 138). DunedinPACE: Intrinsic pace of biological aging. ageAccelskinbloodHorvath: Intrinsic EAA, calculated for the skinbloodHorvath clock. ageAccelHannum: Intrinsic EAA, calculated for the Hannum clock. ageAccelWu: Intrinsic EAA, calculated for the Wu clock. Models were adjusted for adolescent age, race, maternal education, asthma diagnosis, ADHD diagnosis, anxiety symptoms, serum cotinine concentrations, physical activity, BMI *z*-score, and HEI-2010 scores.

We did not find that the associations between sleep efficiency or number of awakenings and EA measures differed by sex (Table S4; Figures 4 and 5). However, we did find sex differences in the associations of total sleep time and sleep fragmentation with several outcomes (interaction *p*-values ranging from .005 to .047; Table S4). Among males, we found a positive trend between total sleep time and the pace of biological aging (*β* = 0.260; 95% CI = –0.029, 0.549), while the corresponding estimate among females was in the opposite direction and similarly imprecise (*β* = –0.173; 95% CI = –0.429, 0.083; sex-by-sleep interaction term *p*-value = .047). Among males, higher sleep fragmentation was also associated with EAA based on the Wu clock (*β* = 0.307; 95% CI = 0.040, 0.574) and the Hannum clock (*β* = 0.885; 95% CI = 0.132, 1.638). The association between sleep fragmentation and EAA based on the skinbloodHorvath clock was directionally consistent but less precise and included the null (*β* = 0.637; 95% CI = –0.217, 1.490), suggesting a coherent positive pattern across clocks. These associations were largely null among females (Wu *β* = –0.079; 95% CI = –0.263, 0.105; Hannum *β* = –0.325; 95% CI = –0.866, 0.217; skinbloodHorvath *β* = –0.175; 95% CI = –0.747, 0.398).

### Sensitivity analyses

In sensitivity analyses removing influential observations, associations between adolescent sleep and epigenetic aging remained consistent with the primary findings (Figures S4 and S5). Furthermore, excluding BMI *z*-scores from the covariate set did not meaningfully change our results, the magnitude of associations remained small and CIs spanned the null (Figures S6 and S7). Results from sex-stratified analyses adjusting for pubertal development were also generally similar to the main results (Table S5).

## Discussion

Overall, we did not find consistent evidence of associations between sleep measures and epigenetic aging in preliminary analyses of the full cohort. In analyses among males only, we found evidence suggesting that total sleep time was positively associated with the pace of biological aging, while we noted negative trends among females. We also found evidence among males of positive associations between sleep fragmentation and EA acceleration across first generation clocks; these associations were attenuated among females. Our findings suggest that associations between sleep measures and epigenetic aging may be subtle, as well as sex- and clock-specific during this critical developmental period.

Research linking epigenetic aging to various health outcomes has increased substantially in recent years. An accelerated EA has been associated with an increased risk for cardiometabolic disease, inflammation, cognitive decline, and earlier mortality [8, 47, 48]. Findings from the recent study by Arzu and colleagues [27] support this association among adolescents, showing that faster biological aging, as measured by DunedinPACE, was associated with higher cardiometabolic risk, including elevated visceral adiposity and hemoglobin A1c. While our study did not directly assess cardiometabolic outcomes, our findings contribute to the existing literature by examining whether objectively measured sleep characteristics, a potential modifiable behavioral factor, may be associated with early biological aging.

In addition to studies linking epigenetic aging quantified at a single adolescent timepoint with health outcomes, recent longitudinal evidence indicates that developmental changes in EA between multiple timepoints predict psychosocial outcomes in young adulthood [49]. In a prospective study spanning adolescence to young adulthood, Mastrotheodoros et al. reported that greater increases in EA from 17 to 25 years were associated with more externalizing problems (GrimAge *β* = 0.13, 95% CI: 0.01, 0.25; Hannum *β* = 0.13, 95% CI: 0.01, 0.24; PhenoAge *β* = 0.15, 95% CI: 0.03, 0.26) and reduced self-concept clarity (Horvath *β* = −0.12, 95% CI: −0.24, 0.00). Although our data are cross-sectional at age 12 years and thus cannot directly capture epigenetic aging trajectories, this evidence highlights the potential downstream relevance of epigenetic aging patterns that may begin to emerge during early adolescence.

There are several biological mechanisms through which sleep may influence epigenetic aging, and our findings, though largely imprecise, are consistent with these proposed pathways. Sleep plays a crucial role in maintaining the body’s health by supporting cellular repair, regulating hormones, and balancing metabolism, and actigraphy, captures key metrics including total sleep time, sleep efficiency, sleep fragmentation, and sleep awakenings, all of which are closely tied to these physiological processes [20, 50]. When sleep is consistently disrupted, it can lead to chronic physiological stress, imbalances in neuroendocrine activity, and increased inflammation. These effects are known to contribute to changes in DNA methylation [5, 51]. Studies have shown that fragmented sleep and shorter sleep duration are associated with elevated inflammatory markers and abnormal cortisol patterns [52, 53]. Over time, these disruptions may interfere with gene regulation, DNA repair, and cellular aging, potentially accelerating epigenetic aging.

Our findings from sex-stratified analyses are consistent with those of Jansen et al. [54], who, using an epigenome-wide approach, reported that sleep duration and midpoint were associated with differentially methylated regions primarily among males. Furthermore, this study noted significant pathway enrichment related to metabolic and developmental processes that were specific to males. Females showed comparatively fewer associations and no significantly enriched pathways. Although the two studies differ in their epigenetic constructs, with Jansen et al. [54] examining locus-specific DNA methylation, whereas the present study uses epigenetic clock-based measures of biological aging, both studies found that sleep-related epigenetic associations during adolescence may be more pronounced in males. The exact mechanisms underlying these sex-specific effects of sleep on DNA methylation and biological aging remain to be elucidated. However, these pathways could involve sex-specific pubertal changes in hormones that influence both sleep architecture and DNA methylation [39, 55, 56]. Estrogen, for example, modulates inflammatory signaling and may affect DNA methylation processes in ways that differ by sex [40]. In addition, adolescent males and females can differ in behavioral sleep patterns, stress exposure, or sensitivity to environmental influences, which could shape how sleep relates to the biology of aging [37, 43]. Future longitudinal analyses with larger sample sizes may provide more mechanistic insights into these sex-specific associations by examining the role of pubertal development.

The observed differences in patterns between first-generation clocks and the pace of biological aging, as estimated using DunedinPACE in a sex-stratified analysis, warrant consideration. First-generation clocks, such as Wu, skinbloodHorvath, and Hannum, were trained to predict chronological age [23–25] and may capture cumulative epigenetic changes that accumulate more gradually over development. DunedinPACE, in contrast, reflects the concurrent pace of biological aging rather than deviation from a chronological reference, and it was developed and validated in an adult population [26]. This methodological distinction may partly explain why sex-stratified patterns were more evident in first-generation EAA measures than in DunedinPACE, though we cannot rule out that the difference reflects true biological heterogeneity in how sleep relates to different dimensions of epigenetic aging during early adolescence.

Several limitations should be considered in our study. Since the study was cross-sectional, we cannot determine whether poor sleep contributes to accelerated aging or whether the association works in the opposite direction. Although the 12-year visits were conducted between 2016 and 2019, generalizability to contemporary adolescents may be somewhat limited as sleep behaviors, digital media use, and the broader mental health context may have evolved over time [57–60]. Actigraphy, while useful for estimating sleep and wake states, cannot capture sleep stages or detect specific sleep disorders. A well-documented limitation of wrist-worn actigraphy is its low specificity for wakefulness; while sensitivity for detecting sleep typically exceeds 0.96, specificity for detecting wake averaged only 0.33, because periods of quiet wakefulness with minimal wrist movement are frequently misclassified as sleep, leading to an overestimation of sleep time and an underestimation of wake after sleep onset [61]. It is also possible that device non-wear or missing data affected some of the sleep estimates [62], though we took careful steps to minimize this during data processing. We also did not include self-reported measures of sleep, which might have offered additional context on how adolescents perceive their own sleep quality [63]. Overall, we expect that misclassification of sleep measures could contribute to our imprecise effect estimates.

Another important limitation concerns the epigenetic clocks used. Although the skinbloodHorvath epigenetic clock included pediatric populations in its training datasets, the wide age ranges, covering various developmental stages of childhood and extending into adulthood, may have affected the accuracy of their age estimates and, consequently, their performance in our cohort [23]. Similarly, the Hannum [24] and DunedinPACE [26] clocks were developed exclusively in adult populations, meaning their performance and sensitivity in younger cohorts are not fully established, and may contribute to attenuated or unstable effect estimates in our sample [64].

Our sample size was relatively small, especially in the stratified analyses by sex. This may limit our ability to detect subtle associations or to generalize findings to a broader adolescent population. Furthermore, there is a potential for selection bias, as we excluded adolescents with missing data on the variables of interest. Residual confounding is also possible; we were unable to account for several potentially relevant factors, including behavioral factors, like alcohol or digital media use, chronic stress, environmental exposures, or genetic variation, which may confound the observed relationships. If associated with both sleep and epigenetic aging, these unmeasured factors could have biased the observed associations. Although our study did not include these factors, prior research demonstrates that environmental influences can shape epigenetic aging in complex ways. For instance, Meier et al. [65] found that childhood trauma, particularly physical neglect among formerly institutionalized young adults, was associated with EA deceleration rather than acceleration, highlighting that developmental contexts may yield heterogeneous effects on biological aging.

Our study also has several important strengths. We used actigraphy to provide an objective assessment of sleep patterns over several nights in the participants’ home environments, offering a more accurate and detailed picture of typical sleep in their daily lives. This approach avoids the limitations of self-report and allows for a nuanced understanding of sleep timing, duration, and continuity [11, 20, 50]. We also examined a range of novel epigenetic aging metrics. The application of first-generation epigenetic clocks, which predict chronological age, and DuneinPACE, which reflects the pace of biological aging, allowed us to explore complementary dimensions of the aging process during a developmental period when these biological systems are still maturing [66, 67]. Because EAA reflects the deviation of EA from chronological age, it is particularly informative for our aims, whereas EA provides complementary context regarding absolute biological maturity. Focusing on early adolescence also adds value, as this is a period when both sleep behaviors and biological aging may still be modifiable. The study was also supported by rigorous protocols for data collection and processing, which helped strengthen the internal validity of our findings.

In summary, our findings from these preliminary analyses suggest that during early adolescence, poorer sleep quality, particularly greater sleep fragmentation, may be associated with higher EA acceleration among males, while these associations were attenuated among females. Further research, including larger longitudinal studies, will be critical to understanding how sleep influences biological aging over time. Combining actigraphy with other sleep assessment tools, such as polysomnography or self-reported sleep quality, may provide a more comprehensive understanding of how sleep health affects epigenetic aging. Future studies with larger and more diverse populations will also help clarify whether certain adolescents are more vulnerable to the aging effects of poor sleep.

## Supplementary Material

2026_05_25_Supplemetary_Files_SLEEP_ADVANCES_v2_zpag090

## Data Availability

Data are available upon reasonable request. The HOME study Principal Investigators welcome new collaborations with other investigators and actively engage in collaborative data-sharing projects. Interested investigators should visit https://homestudy.research.cchmc.org/contact or contact KY (kimberly.yolton@cchmc.org) to obtain additional information about The HOME study, discuss collaborative opportunities, and request a project proposal form. The HOME study Protocol Review Committee reviews proposed research projects to ensure that they do not overlap with extant projects and are an efficient use of scarce resources (e.g. biospecimens).
