# Supplementary material for: The association of sleep health with epigenetic aging in early adolescence: exploratory insights from the Health Outcomes and Measures of the Environment study
Source: Sleep Adv. 2026 Aug 13;7(3):zpag090. doi: 10.1093/sleepadvances/zpag090 (PMC13570646; doi:10.1093/sleepadvances/zpag090)
Supplement: Supplementary file 1 [file 2026_05_25_supplemetary_files_sleep_advances_v2_zpag090.docx]

# The Association of Sleep Health with Epigenetic Aging in Early Adolescence: Exploratory Insights from the HOME Study

Smriti Maskey^1, 2^, Jennifer Arzu^3^, Aimin Chen^4^, Karl Kelsey^3^, Scott M. Langevin^5,6^, Kim M. Cecil^7,8,9^, Kimberly Yolton^8,9^, Joseph M. Braun^3^, Clara G. Sears^1^

1. Christina Lee Brown Envirome Institute, University of Louisville School of Medicine, Louisville, KY, USA
2. Department of Epidemiology and Population Health Sciences, University of Louisville School of Public Health and Information Sciences, Louisville, KY, USA
3. Department of Epidemiology, School of Public Health, Brown University, Providence, RI, USA
4. Department of Biostatistics, Epidemiology and Informatics, University of Pennsylvania Perelman School of Medicine, Philadelphia, PA, USA
5. Larner College of Medicine, University of Vermont, Burlington, VT, USA
6. University of Vermont Cancer Center, Burlington, VT, USA
7. Department of Radiology, University of Cincinnati College of Medicine, Cincinnati, OH, USA
8. Department of Environmental and Public Health Sciences, University of Cincinnati College of Medicine, Cincinnati, OH, USA
9. Department of Pediatrics, Cincinnati Children’s Hospital Medical Center, University of Cincinnati College of Medicine, Cincinnati, OH, USA

Corresponding Author: Dr. Clara G. Sears, Christina Lee Brown Envirome Institute, University of Louisville School of Medicine, clara.sears@louisville.edu

**TABLE of Supplemental Tables**

[Supplemental Table 1: ANOVA Comparison: Spline vs Linear Models for Total Sleep Time with EA, EAA, and a pace of biological aging at 12 years (the HOME Study; Cincinnati, OH; n=138)	5](#_Toc236920475)

[Supplemental Table 2: Comparison of included and excluded participant characteristics in complete case analysis (N=242, the HOME Study, Cincinnati, OH, 2003-2019)	6](#_Toc236920476)

[Supplemental Table 3: Participant Characteristics by epigenetic age acceleration measures and pace of biological aging at 12 years of age in complete case analysis (n = 138, the HOME Study, Cincinnati, OH, 2003-2019)	8](#_Toc236920477)

[Supplemental Table 4: Sex-stratified associations of sleep measures with epigenetic age, epigenetic age acceleration, and the pace of biological aging at 12 years (n = 138, the HOME Study, Cincinnati, OH, 2003-2019)	10](#_Toc236920478)

[Supplemental Table 5: Sex-stratified association in epigenetic age clocks per standard deviation increase in sleep measures, adjusted for pubertal development among adolescents (n = 138, the HOME Study, Cincinnati, OH, 2003-2019)	16](#_Toc236920479)

**TABLE OF SUPPLEMENTAL FIGURES**

[Supplemental Figure 1: Flowchart of missing data and selection of participants for complete case analyses (HOME Study, OH; 2003-2019)	2](#_Toc236920717)

[Supplemental Figure 2: Directed Acyclic Graph (DAG) for association between sleep measures and epigenetic age outcomes	3](#_Toc236920718)

[Supplemental Figure 3: Exploratory Spline Effects of Total Sleep Time on EAAs and the pace of biological aging*.*	5](#_Toc236920719)

[Supplemental Figure 4: Adjusted difference (95% CI) in epigenetic clocks per standard deviation increase in sleep measures among adolescents, after removal of outliers (the HOME Study, Cincinnati, OH, 2003-2019).	13](#_Toc236920720)

[Supplemental Figure 5: Adjusted difference (95% CI) in epigenetic age acceleration per standard deviation increase in sleep measures among adolescents, after removal of outliers (the HOME Study, Cincinnati, OH, 2003-2019).	14](#_Toc236920721)

[Supplemental Figure 6: Adjusted difference (95% CI) in epigenetic clocks per standard deviation increase in sleep measures among adolescents, after removal of Body Mass Index (the HOME Study, Cincinnati, OH, 2003-2019).	15](#_Toc236920722)

[Supplemental Figure 7: Adjusted estimates (95% CI) in epigenetic age acceleration per standard deviation increase in sleep measures among adolescents, after removal of Body Mass Index (the HOME Study, Cincinnati, OH, 2003-2019).	16](#_Toc236920723)

Supplemental Figure 1: Flowchart of missing data and selection of participants for complete case analyses (HOME Study, OH; 2003-2019)

**Supplemental Figure 2:** Directed Acyclic Graph (DAG) for association between sleep measures and epigenetic age outcomes

## Supplemental Methods: Exploratory Spline Analysis of Total Sleep Time

We examined spline effects specifically for total sleep time because prior literature has most consistently reported **nonlinear (often U-shaped) associations** between **sleep duration** and a variety of health outcomes, including **mortality, cardiovascular disease, metabolic dysfunction,** and **cognitive aging**. These patterns suggest that both short and long sleep durations may be linked to adverse biological outcomes.^1,2^ In contrast, other sleep metrics, such as sleep efficiency, number of awakenings, and fragmentation index, are generally considered linear indicators of sleep quality. Given this difference in established biological patterns, we restricted our exploratory spline analyses to total sleep time, where nonlinearity was both **biologically plausible** and **empirically supported** by prior research.

As shown in Supplemental Figure 3, the predicted relationships between sleep duration and epigenetic age accelerations calculated using first-generation epigenetic clocks (Wu, skinbloodHorvath, and Hannum) and pace of biological aging were generally curvilinear, with several models suggesting a slight inverted U-shape- indicating that both shorter and longer sleep durations may be associated with lower epigenetic age acceleration or pace of biological aging at 12 years, though patterns varied across clocks.

However, tests of non-linearity using ANOVA (comparing spline models to linear models) indicated that none of the spline terms were statistically significant (all p>0.15) (Supplementary Table 1). These findings suggest limited evidence for meaningful non-linear associations between total sleep time and EA, EAA and a pace of biological aging at 12 years in this cohort.

**Supplemental Figure 3:** Exploratory Spline Effects of Total Sleep Time on EAAs and the pace of biological aging*.*

Abbreviations note: DunedinPACE: Intrinsic pace of biological aging; ageAccelskinbloodHorvath: Intrinsic Epigenetic Age Acceleration, calculated for the skinbloodHorvath clock; ageAccelHannum: Intrinsic Epigenetic Age Acceleration, calculated for the Hannum clock; ageAccelWu: Intrinsic Epigenetic Age Acceleration, calculated for the Wu clock.

|  | **Supplemental Table 1:** ANOVA Comparison: Spline vs Linear Models for Total Sleep Time with EA, EAA, and a pace of biological aging at 12 years (the HOME Study; Cincinnati, OH; n=138) | | | | |
| --- | --- | --- | --- | --- | --- |
|  | **Outcome** | **df^a^** | **Sum of Squares** | **F-value** | **Pr (>F)^a^** |
| Wu | | 2 | 0.72 | 0.31 | 0.73 |
| Hannum | | 2 | 23.40 | 0.79 | 0.46 |
| skinbloodHorvath | | 2 | 25.09 | 1.09 | 0.34 |
|  | ageAccelWu | 2 | 0.53 | 0.37 | 0.69 |
|  | ageAccelHannum | 2 | 17.76 | 1.50 | 0.23 |
|  | ageAccelskinbloodHorvath | 2 | 22.30 | 1.70 | 0.19 |
|  | DunedinPACE | 2 | 1.75 | 1.10 | 0.33 |
|  | **^a^Pr (>F)** is the p-value of the F-statistics. Abbreviations note: df: degree of freedom; DunedinPACE: Intrinsic pace of biological aging; ageAccelskinbloodHorvath: Intrinsic Epigenetic Age Acceleration, calculated for the skinbloodHorvath clock; ageAccelHannum: Intrinsic Epigenetic Age Acceleration, calculated for the Hannum clock; ageAccelWu: Intrinsic Epigenetic Age Acceleration, calculated for the Wu clock. | | | | |

| **Supplemental Table 2:** Comparison of included and excluded participant characteristics in complete case analysis (N=242, the HOME Study, Cincinnati, OH, 2003-2019) | | | | |  |
| --- | --- | --- | --- | --- | --- |
| **Category** | **Excluded [n(%)]** | **Included [n(%)]** | | p-value^a^ |  |
| **Overall** | **104 (42.9)** | **138 (57.1)** |  | |  |
| **Adolescent Sex** |  |  | 0.358 | |  |
| Female | 54 (51.9) | 81 (58.7) |  | |  |
| Male | 50 (48.1) | 57 (41.3) |  | |  |
| **Adolescent Race** |  |  | 0.659 | |  |
| White | 57 (54.8) | 80 (58.0) |  | |  |
| Black or African American | 40 (38.5) | 46 (33.3) |  | |  |
| Other/Multiracial^b^ | 7 ( 6.7) | 12 ( 8.7) |  | |  |
| **Age (Years, in Tertiles)** |  |  | 0.031 | |  |
| 11.05 to 12.03 | 13 (26.0) | 50 (36.2) |  | |  |
| 12.03 to 12.65 | 13 (26.0) | 50 (36.2) |  | |  |
| 12.65 to 14.09 | 24 (48.0) | 38 (27.5) |  | |  |
| **Mother’s Education** |  |  | 0.924 | |  |
| College Graduate or Above | 52 (51.0) | 73 (52.9) |  | |  |
| High School and Some College | 43 (42.2) | 57 (41.3) |  | |  |
| Less than High School | 7 ( 6.9) | 8 ( 5.8) |  | |  |
| **Asthma Diagnosis, yes** | 25 (24.0) | 31 (22.5) | 0.894 | |  |
| **ADHD Diagnosis, yes** | 20 (19.4) | 25 (18.1) | 0.929 | |  |
| **Adolescent Serum Cotinine Concentration (ng/mL)** |  |  | 0.512 | |  |
| < 0.04 | 19 (33.3) | 46 (33.3) |  | |  |
| 0.04 to 0.08 | 16 (28.1) | 49 (35.5) |  | |  |
| > 0.08 | 22 (38.6) | 43 (31.2) |  | |  |
| **Body Mass Index (BMI)^c^** |  |  | 0.495 | |  |
| Underweight | 2 ( 1.9) | 3 ( 2.2) |  | |  |
| Normal weight | 70 (68.0) | 83 (60.1) |  | |  |
| Overweight | 23 (22.3) | 43 (31.2) |  | |  |
| Obesity | 8 ( 7.8) | 9 ( 6.5) |  | |  |
| **Anxiety^d^,** Elevated (> 60) | 19 (18.6) | 21 (15.2) | 0.599 | |  |
| **Physical Activity^e^** |  |  | 0.450 | |  |
| Inactive | 9 (14.5) | 17 (12.3) |  | |  |
| Meets Minimum Recommendation | 29 (46.8) | 51 (37.0) |  | |  |
| Above Recommendation | 14 (22.6) | 44 (31.9) |  | |  |
| Highly Active | 10 (16.1) | 26 (18.8) |  | |  |
| **Healthy Eating Index^f^** |  |  | 0.627 | |  |
| < 39.84 | 34 (34.7) | 45 (32.6) |  | |  |
| 39.84 to 48.88 | 35 (35.7) | 44 (31.9) |  | |  |
| > 48.88 | 29 (29.6) | 49 (35.5) |  | |  |
| Note: HOME, Health Outcomes and Measures of the Environment; SD, standard deviation.  ADHD: Attention-deficit hyperactivity disorder  ^a^ Wilcoxon rank sum test; Pearson's Chi-squared test; Fisher's exact test  ^b^Other/Multiracial includes participants identifying as Asian/Pacific Islander or American Indian  ^c^Body Mass Index (BMI) was measured as a z-score. Categories are defined as: Underweight (z < -2), Normal weight (-2 ≤ z < 1), Overweight (1 ≤ z < 2), and Obesity (z ≥ 2).  ^d^Anxiety level was assessed using Total Scores from the Spence Children’s Anxiety Scale (range: 1-100). Elevated anxiety was defined as a score ≥ 60; normal anxiety was defined as a score < 60.  ^e^Physical activity represents the average daily minutes of moderate-to-vigorous physical activity at age 12, measured by wrist-worn accelerometers. Categories are defined as: Inactive (< 60 min/day), Meets minimum recommendation (60-90 min/day), Above recommendation (90-120 min/day), and Highly active (≥ 120 min/day).  ^f^Healthy Eating Index (HEI) scores were based on the HEI-2010 (total possible score: 100), derived from food frequency questionnaire data | | | | |  |

**Supplemental Table 3:** Participant Characteristics by epigenetic age acceleration measures and pace of biological aging at 12 years of age in complete case analysis (n = 138, the HOME Study, Cincinnati, OH, 2003-2019)

| **Category** | **Total [n(%)]** | **Epigenetic Age Accelerations**  **(mean± SD)** | | | **DunedinPACE^a^**  **(mean± SD)** | |
| --- | --- | --- | --- | --- | --- | --- |
|  |  | **ageAccelWu^a^** | **ageAccel**  **skinbloodHorvath^a^** | **ageAccel**  **Hannum^a^** |  |  |
| **Overall** | **138(100)** | **-0.06 ± 0.87** | **-0.11 ± 2.72** | **-0.24 ± 2.63** | **-0.00 ± 0.98** |  |
| **Adolescent Sex** |  |  |  |  |  |  |
| Female | 81 (58.7) | -0.03 ± 0.83 | -0.07 ± 2.67 | -0.37 ± 2.64 | -0.16 ± 0.96 |  |
| Male | 57 (41.3) | -0.11 ± 0.93 | -0.17 ± 2.81 | -0.05 ± 2.64 | 0.22 ± 0.97 |  |
| **Adolescent Race** |  |  |  |  |  |  |
| White | 80 (58.0) | 0.11 ± 0.89 | 0.12 ± 2.81 | 0.08 ± 2.64 | -0.26 ± 0.76 |  |
| Black or African American | 46 (33.3) | -0.37 ± 0.82 | -0.54 ± 2.76 | -0.83 ± 2.72 | 0.41 ± 1.22 |  |
| Other/Multiracial^b^ | 12 (8.7) | 0.02 ± 0.65 | -0.03 ± 1.73 | -0.13 ± 1.95 | 0.20 ± 0.67 |  |
| **Age (Years, in Tertiles)** |  |  |  |  |  |  |
| 11.05 to 12.03 | 50 (36.2) | -0.01 ± 0.90 | 0.22 ± 2.93 | 0.08 ± 2.65 | -0.15 ± 1.03 |  |
| 12.03 to 12.65 | 50 (36.2) | -0.12 ± 0.81 | -0.49 ± 2.40 | -0.47 ± 2.48 | 0.05 ± 0.85 |  |
| 12.65 to 14.09 | 38 (27.5) | -0.05 ± 0.94 | -0.06 ± 2.84 | -0.36 ± 2.82 | 0.12 ± 1.07 |  |
| **Mother’s Education** | | |  |  |  |  |
| College Graduate or Above | 73 (52.9) | 0.09 ± 0.85 | 0.07 ± 2.60 | -0.05 ± 2.69 | -0.09 ± 0.92 |  |
| High School and Some College | 57 (41.3) | -0.28 ± 0.83 | -0.72 ± 2.42 | -0.77 ± 2.37 | 0.15 ± 1.02 |  |
| Less than High School | 8 (5.8) | 0.14 ± 1.15 | 2.54 ± 4.10 | 1.83 ± 2.94 | -0.30 ± 1.17 |  |
| **Asthma Diagnosis** |  |  |  |  |  |  |
| No | 107 (77.5) | -0.03 ± 0.86 | -0.00 ± 2.63 | -0.13 ± 2.51 | -0.01 ± 0.95 |  |
| Yes | 31 (22.5) | -0.17 ± 0.94 | -0.50 ± 3.01 | -0.62 ± 3.01 | 0.03 ± 1.08 |  |
| **ADHD Diagnosis** |  |  |  |  |  |  |
| No | 113 (81.9) | 0.01 ± 0.88 | 0.15 ± 2.81 | -0.03 ± 2.61 | -0.04 ± 0.90 |  |
| Yes | 25 (18.1) | -0.40 ± 0.76 | -1.32 ± 1.89 | -1.21 ± 2.58 | 0.20 ± 1.29 |  |
| **Adolescent Serum Cotinine Concentration (ng/mL)** | | | | |  |  |
| < 0.04 | 46 (33.3) | 0.11 ± 0.91 | 0.04 ± 2.89 | -0.03 ± 2.79 | -0.17 ± 0.94 |  |
| 0.04 to 0.08 | 49 (35.5) | -0.01 ± 0.82 | 0.13 ± 2.52 | 0.04 ± 2.41 | -0.02 ± 0.75 |  |
| > 0.08 | 43 (31.2) | -0.30 ± 0.86 | -0.55 ± 2.76 | -0.79 ± 2.67 | 0.21 ± 1.20 |  |
| **Body Mass Index (BMI)^c^** | |  |  |  |  |  |
| Underweight | 3 (2.2) | 0.05 ± 0.74 | 0.11 ± 2.59 | 0.22 ± 2.05 | 0.96 ± 1.83 |  |
| Normal weight | 83 (60.1) | 0.04 ± 0.92 | 0.16 ± 3.00 | 0.18 ± 2.62 | -0.08 ± 0.80 |  |
| Overweight | 43 (31.2) | -0.19 ± 0.80 | -0.42 ± 2.07 | -0.83 ± 2.42 | -0.06 ± 1.13 |  |
| Obesity | 9 (6.5) | -0.43 ± 0.78 | -1.28 ± 2.67 | -1.43 ± 3.30 | 0.68 ± 1.22 |  |
| **Anxiety^d^** |  |  |  |  |  |  |
| Normal (<60) | 117 (84.8) | 0.01 ± 0.85 | 0.04 ± 2.80 | -0.03 ± 2.65 | -0.01 ± 1.01 |  |
| Elevated (60 and above) | 21 (15.2) | -0.48 ± 0.89 | -0.94 ± 2.09 | -1.39 ± 2.25 | 0.05 ± 0.81 |  |
| **Physical Activity^e^** |  |  |  |  |  |  |
| Inactive | 17 (12.3) | -0.15 ± 0.72 | -0.59 ± 1.93 | -0.37 ± 2.39 | -0.01 ± 0.82 |  |
| Meets Minimum Recommendation | 51 (37.0) | 0.00 ± 0.82 | 0.12 ± 2.29 | 0.17 ± 2.64 | -0.02 ± 1.06 |  |
| Above Recommendation | 44 (31.9) | 0.05 ± 1.06 | 0.26 ± 3.62 | -0.35 ± 2.83 | -0.11 ± 0.89 |  |
| Highly Active | 26 (18.8) | -0.31 ± 0.69 | -0.90 ± 1.96 | -0.77 ± 2.45 | 0.23 ± 1.07 |  |
| **Healthy Eating Index^f^** | |  |  |  |  |  |
| < 39.84 | 45 (32.6) | -0.25 ± 0.75 | -0.79 ± 1.98 | -0.99 ± 2.46 | 0.24 ± 1.11 |  |
| 39.84 to 48.88 | 44 (31.9) | -0.08 ± 0.87 | 0.02 ± 2.81 | -0.07 ± 2.75 | -0.21 ± 0.78 |  |
| > 48.88 | 49 (35.5) | 0.13 ± 0.96 | 0.39 ± 3.12 | 0.30 ± 2.56 | -0.03 ± 0.98 |  |
| **Sleep Efficiency (%)** | |  |  |  |  |  |
| 76.16 to 86.68 | 43 (31.2) | 0.13 ± 0.90 | 0.31 ± 2.37 | 0.51 ± 2.23 | -0.14 ± 0.81 |  |
| 86.68 to 90.32 | 47 (34.1) | -0.08 ± 0.90 | 0.10 ± 3.19 | -0.33 ± 2.81 | 0.22 ± 0.99 |  |
| 90.32 to 96.39 | 48 (34.8) | -0.21 ± 0.81 | -0.70 ± 2.45 | -0.83 ± 2.67 | -0.08 ± 1.08 |  |
| **Total Sleep Time (minutes)** | | | | | | |
| 247.20 to 402.39 | 43 (31.2) | -0.05 ± 0.93 | 0.08 ± 2.59 | -0.06 ± 2.61 | -0.02 ± 0.94 |  |
| 402.39 to 446.71 | 45 (32.6) | -0.17 ± 0.78 | -0.32 ± 2.66 | -0.23 ± 2.58 | 0.04 ± 1.04 |  |
| 446.71 to 565.33 | 50 (36.2) | 0.02 ± 0.91 | -0.09 ± 2.91 | -0.40 ± 2.74 | -0.02 ± 0.97 |  |
| **Number of Awakenings** | | |  |  |  |  |
| 17.07 to 22.98 | 49 (35.5) | 0.09 ± 0.96 | 0.34 ± 3.18 | 0.00 ± 2.66 | -0.08 ± 0.92 |  |
| 22.98 to 32.83 | 44 (31.9) | 0.03 ± 0.75 | -0.15 ± 2.00 | 0.02 ± 2.44 | -0.07 ± 0.86 |  |
| 6.60 to 17.07 | 45 (32.6) | -0.32 ± 0.84 | -0.57 ± 2.76 | -0.76 ± 2.77 | 0.15 ± 1.14 |  |
| **Sleep Fragmentation Index (%)** | | | | | | |
| 19.45 to 23.11 | 47 (34.1) | 0.03 ± 0.77 | -0.06 ± 2.55 | -0.06 ± 2.38 | -0.11 ± 1.00 |  |
| 23.11 to 37.05 | 44 (31.9) | -0.03 ± 0.93 | 0.29 ± 2.89 | 0.15 ± 2.73 | 0.14 ± 0.92 |  |
| 6.55 to 19.45 | 47 (34.1) | -0.18 ± 0.92 | -0.54 ± 2.70 | -0.79 ± 2.74 | -0.02 ± 1.01 |  |
| *Note:* HOME, Health Outcomes and Measures of the Environment; SD, standard deviation.  ADHD: Attention deficit hyperactivity disorder  ^a^Intrinsic epigenetic measures are reported (i.e., adjusted for cell type proportions).  ^b^Other/Multiracial includes participants identifying as Asian/Pacific Islander or American Indian  ^c^Body Mass Index (BMI) was measured as a z-score. Categories are defined as: Underweight (z < -2), Normal weight (-2 ≤ z < 1), Overweight (1 ≤ z < 2), and Obesity (z ≥ 2).  ^d^Anxiety level was assessed using Total Scores from the Spence Children’s Anxiety Scale (range: 1-100). Elevated anxiety was defined as a score ≥ 60; normal anxiety was defined as a score < 60.  ^e^Physical activity represents the average daily minutes of moderate-to-vigorous physical activity at age 12, measured by wrist-worn accelerometers. Categories are defined as: Inactive (< 60 min/day), Meets minimum recommendation (60-90 min/day), Above recommendation (90-120 min/day), and Highly active (≥ 120 min/day).  ^f^Healthy Eating Index (HEI) scores were based on the HEI-2010 (total possible score: 100), derived from food frequency questionnaire data. | | | | | | |

| **Supplemental Table 4:** Sex-stratified associations of sleep measures with epigenetic age, epigenetic age acceleration, and the pace of biological aging at 12 years (n = 138, the HOME Study, Cincinnati, OH, 2003-2019) | | | | |
| --- | --- | --- | --- | --- |
| **Sleep Variable** | **Outcome** | **Male**  **(Beta 95% CI)** | **Female**  **(Beta 95% CI)** | **P-Interaction** |
| Sleep Efficiency | Wu | -0.468  (-0.862, -0.073) | 0.001  (-0.230, 0.232) | 0.152 |
|  | Hannum | -1.595  (-2.852, -0.337) | 0.240  (-0.680, 1.159) | 0.115 |
|  | skinbloodHorvath | -1.065  (-2.312, 0.182) | 0.062  (-0.714, 0.838) | 0.275 |
|  | ageAccelWu | -0.252  (-0.544, 0.040) | -0.003  (-0.186, 0.180) | 0.137 |
|  | ageAccelHannum | -0.636  (-1.468, 0.196) | -0.051  (-0.592, 0.490) | 0.238 |
|  | ageAccelskinbloodHorvath | -0.484  (-1.406, 0.438) | 0.062  (-0.505, 0.628) | 0.196 |
|  | DunedinPACE | 0.095  (-0.223, 0.413) | -0.106  (-0.311, 0.099) | 0.11 |
| Total Sleep Time | Wu | 0.083  (-0.310, 0.477) | -0.118  (-0.406, 0.171) | 0.139 |
|  | Hannum | -0.604  (-1.858, 0.650) | 0.098  (-1.059, 1.256) | 0.94 |
|  | skinbloodHorvath | 0.151  (-1.059, 1.361) | -0.292  (-1.264, 0.680) | 0.1 |
|  | ageAccelWu | 0.060  (-0.223, 0.344) | -0.014  (-0.244, 0.216) | 0.239 |
|  | ageAccelHannum | -0.355  (-1.151, 0.440) | 0.080  (-0.599, 0.759) | 0.836 |
|  | ageAccelskinbloodHorvath | 0.021  (-0.856, 0.899) | -0.083  (-0.794, 0.629) | 0.168 |
|  | DunedinPACE | 0.260  (-0.029, 0.549) | -0.173  (-0.429, 0.083) | 0.047* |
| Number of Awakenings | Wu | 0.424  (0.014, 0.834) | -0.046  (-0.298, 0.205) | 0.128 |
|  | Hannum | 0.927  (-0.426, 2.281) | -0.338  (-1.340, 0.664) | 0.221 |
|  | skinbloodHorvath | 0.695  (-0.609, 1.999) | -0.249  (-1.094, 0.595) | 0.183 |
|  | ageAccelWu | 0.227  (-0.075, 0.529) | 0.020  (-0.179, 0.220) | 0.136 |
|  | ageAccelHannum | 0.376  (-0.492, 1.244) | 0.016  (-0.574, 0.606) | 0.14 |
|  | ageAccelskinbloodHorvath | 0.214  (-0.741, 1.169) | -0.028  (-0.646, 0.590) | 0.146 |
|  | DunedinPACE | 0.144  (-0.180, 0.469) | 0.028  (-0.197, 0.253) | 0.792 |
| Sleep Fragmentation Index | Wu | 0.382  (0.007, 0.758) | 0.032  (-0.202, 0.265) | 0.231 |
|  | Hannum | 1.466  (0.284, 2.649) | -0.036  (-0.968, 0.897) | 0.112 |
|  | skinbloodHorvath | 0.856  (-0.324, 2.036) | 0.242  (-0.541, 1.025) | 0.493 |
|  | ageAccelWu | 0.307  (0.040, 0.574) | -0.079  (-0.263, 0.105) | 0.021* |
|  | ageAccelHannum | 0.885  (0.132, 1.638) | -0.325  (-0.866, 0.217) | 0.005* |
|  | ageAccelskinbloodHorvath | 0.637  (-0.217, 1.490) | -0.175  (-0.747, 0.398) | 0.047* |
|  | DunedinPACE | -0.054  (-0.353, 0.245) | 0.023  (-0.186, 0.232) | 0.513 |
| Note:  ageAccelWu: Intrinsic Epigenetic Age Acceleration, derived from the Wu clock, adjusted for cell type proportion  ageAccelskinbloodHorvath: Intrinsic Epigenetic Age Acceleration, derived from the skinbloodHorvath clock, adjusted for cell type proportion  ageAccelHannum: Intrinsic Epigenetic Age Acceleration, derived from the skinbloodHorvath clock, adjusted for cell type proportion  DunedinPACE: Intrinsic pace of biological ageing, adjusted for cell type proportion  Models were adjusted for adolescent age, sex, race, maternal education, asthma diagnosis, ADHD diagnosis, anxiety symptoms, serum cotinine concentrations, physical activity, BMI, and Healthy Eating Index-2010 scores  *Significant at p<0.05 | | | | |

**Supplemental Figure 4:** Adjusted difference (95% CI) in epigenetic clocks per standard deviation increase in sleep measures among adolescents, after removal of outliers (the HOME Study, Cincinnati, OH, 2003-2019).

Models were adjusted for adolescent age, sex, race, maternal education, asthma diagnosis, ADHD diagnosis, anxiety symptoms, serum cotinine concentrations, physical activity, BMI z-score, and Healthy Eating Index-2010 scores

**Supplemental Figure 5:** Adjusted difference (95% CI) in epigenetic age acceleration per standard deviation increase in sleep measures among adolescents, after removal of outliers (the HOME Study, Cincinnati, OH, 2003-2019).

Models were adjusted for adolescent age, sex, race, maternal education, asthma diagnosis, ADHD diagnosis, anxiety symptoms, serum cotinine concentrations, physical activity, BMI z-score, and Healthy Eating Index-2010 scores.

Abbreviations note: DunedinPACE: Intrinsic pace of biological aging; ageAccelskinbloodHorvath: Intrinsic Epigenetic Age Acceleration, calculated for the skinbloodHorvath clock; ageAccelHannum: Intrinsic Epigenetic Age Acceleration, calculated for the Hannum clock; ageAccelWu: Intrinsic Epigenetic Age Acceleration, calculated for the Wu clock.

**Supplemental Figure 6:** Adjusted difference (95% CI) in epigenetic clocks per standard deviation increase in sleep measures among adolescents, after removal of Body Mass Index (the HOME Study, Cincinnati, OH, 2003-2019).

Models were adjusted for adolescent age, sex, race, maternal education, asthma diagnosis, ADHD diagnosis, anxiety symptoms, serum cotinine concentrations, physical activity, and Healthy Eating Index-2010 scores.

**Supplemental Figure 7**: Adjusted estimates (95% CI) in epigenetic age acceleration per standard deviation increase in sleep measures among adolescents, after removal of Body Mass Index (the HOME Study, Cincinnati, OH, 2003-2019).

Models were adjusted for adolescent age, sex, race, maternal education, asthma diagnosis, ADHD diagnosis, anxiety symptoms, serum cotinine concentrations, physical activity, and Healthy Eating Index-2010 scores. Abbreviations note: DunedinPACE: Intrinsic pace of biological aging; ageAccelskinbloodHorvath: Intrinsic Epigenetic Age Acceleration, calculated for the skinbloodHorvath clock; ageAccelHannum: Intrinsic Epigenetic Age Acceleration, calculated for the Hannum clock; ageAccelWu: Intrinsic Epigenetic Age Acceleration, calculated for the Wu clock.

**Supplemental Table 5:** Sex-stratified association in epigenetic age clocks per standard deviation increase in sleep measures, adjusted for pubertal development among adolescents (n = 138, the HOME Study, Cincinnati, OH, 2003-2019)

| **Sleep Variable** | **Outcome** | **Male**  **(Beta 95% CI)** | **Female**  **(Beta 95% CI)** | **P-Interaction** |
| --- | --- | --- | --- | --- |
| Sleep Efficiency | Wu | -0.481  (-0.871, -0.090) | 0.006  (-0.231, 0.244) | 0.142 |
|  | Hannum | -1.626  (-2.883, -0.369) | 0.254  (-0.699, 1.207) | 0.119 |
|  | skinbloodHorvath | -1.100  (-2.343, 0.143) | 0.051  (-0.749, 0.852) | 0.286 |
|  | ageAccelWu | -0.262  (-0.551, 0.028) | -0.002  (-0.191, 0.188) | 0.138 |
|  | ageAccelHannum | -0.642  (-1.484, 0.200) | -0.052  (-0.607, 0.503) | 0.267 |
|  | ageAccelskinbloodHorvath | -0.509  (-1.429, 0.411) | 0.040  (-0.541, 0.621) | 0.224 |
|  | DunedinPACE | 0.098  (-0.224, 0.420) | -0.101  (-0.309, 0.106) | 0.115 |
| Total Sleep Time | Wu | 0.085  (-0.306, 0.477) | -0.121  (-0.426, 0.184) | 0.173 |
|  | Hannum | -0.600  (-1.858, 0.657) | 0.108  (-1.124, 1.340) | 0.918 |
|  | skinbloodHorvath | 0.155  (-1.053, 1.364) | -0.340  (-1.370, 0.690) | 0.123 |
|  | ageAccelWu | 0.062  (-0.220, 0.343) | -0.015  (-0.260, 0.229) | 0.273 |
|  | ageAccelHannum | -0.355  (-1.159, 0.450) | 0.116  (-0.599, 0.832) | 0.812 |
|  | ageAccelskinbloodHorvath | 0.025  (-0.851, 0.901) | -0.123  (-0.872, 0.627) | 0.185 |
|  | DunedinPACE | 0.260  (-0.033, 0.552) | -0.160  (-0.426, 0.107) | 0.039* |
| Number of Awakenings | Wu | 0.522  (0.111, 0.932) | -0.056  (-0.314, 0.203) | 0.103 |
|  | Hannum | 1.151  (-0.236, 2.537) | -0.346  (-1.383, 0.691) | 0.210 |
|  | skinbloodHorvath | 0.927  (-0.404, 2.258) | -0.271  (-1.141, 0.599) | 0.155 |
|  | ageAccelWu | 0.292  (-0.012, 0.597) | 0.016  (-0.190, 0.223) | 0.116 |
|  | ageAccelHannum | 0.426  (-0.479, 1.331) | 0.039  (-0.566, 0.644) | 0.151 |
|  | ageAccelskinbloodHorvath | 0.367  (-0.613, 1.346) | -0.032  (-0.665, 0.602) | 0.127 |
|  | DunedinPACE | 0.137  (-0.202, 0.476) | 0.040  (-0.187, 0.268) | 0.720 |
| Sleep Fragmentation Index | Wu | 0.405  (0.033, 0.777) | 0.019  (-0.223, 0.262) | 0.187 |
|  | Hannum | 1.522  (0.340, 2.704) | -0.029  (-1.004, 0.945) | 0.107 |
|  | skinbloodHorvath | 0.915  (-0.263, 2.092) | 0.203  (-0.612, 1.018) | 0.428 |
|  | ageAccelWu | 0.324  (0.059, 0.588) | -0.088  (-0.281, 0.104) | 0.017* |
|  | ageAccelHannum | 0.898  (0.134, 1.662) | -0.299  (-0.861, 0.262) | 0.006* |
|  | ageAccelskinbloodHorvath | 0.680  (-0.171, 1.531) | -0.207  (-0.797, 0.384) | 0.037* |
|  | DunedinPACE | -0.059  (-0.362, 0.244) | 0.048  (-0.165, 0.260) | 0.462 |
| Note:  ageAccelWu: Intrinsic Epigenetic Age Acceleration, derived from the Wu clock, adjusted for cell type proportion  ageAccelskinbloodHorvath: Intrinsic Epigenetic Age Acceleration, derived from the skinbloodHorvath clock, adjusted for cell type proportion  ageAccelHannum: Intrinsic Epigenetic Age Acceleration, derived from the skinbloodHorvath clock, adjusted for cell type proportion  DunedinPACE: Intrinsic pace of biological aging, adjusted for cell type proportion  *Significant at p<0.05 | | | | |
